# Attenuation properties of poly methyl methacrylate reinforced with micro/nano ZrO_2_ as gamma-ray shields

**DOI:** 10.1038/s41598-024-51551-4

**Published:** 2024-01-13

**Authors:** Mahmoud T. Alabsy, Mahmoud I. Abbas, Alaa Y. El-khatib, Ahmed M. El-Khatib

**Affiliations:** Physics Department, Faculty of Science, Alexandria University, Alexandria, 21511 Egypt

**Keywords:** Nanoscience and technology, Physics

## Abstract

This research aimed to examine the radiation shielding properties of unique polymer composites for medical and non-medical applications. For this purpose, polymer composites, based on poly methyl methacrylate (PMMA) as a matrix, were prepared and reinforced with micro- and nanoparticles of ZrO_2_ fillers at a loading of 15%, 30%, and 45% by weight. Using the high purity germanium (HPGe) detector, the suggested polymer composites’ shielding characteristics were assessed for various radioactive sources. The experimental values of the mass attenuation coefficients (MAC) of the produced composites agreed closely with those obtained theoretically from the XCOM database. Different shielding parameters were estimated at a broad range of photon energies, including the linear attenuation coefficient (μ), tenth value layer (TVL), half value layer (HVL), mean free path (MFP), effective electron density (N_eff_), effective atomic number (Z_eff_), and equivalent atomic number (Z_eq_), as well as exposure buildup factor (EBF) and energy absorption buildup factor (EABF) to provide more shielding information about the penetration of γ-rays into the chosen composites. The results showed that increasing the content of micro and nano ZrO_2_ particles in the PMMA matrix increases μ values and decreases HVL, TVL, and MFP values. P-45nZ sample with 45 wt% of ZrO_2_ nanoparticles had the highest μ values, which varied between 2.6546 and 0.0991 cm^−1^ as γ-ray photon energy increased from 0.0595 to 1.408 MeV, respectively. Furthermore, the highest relative increase rate in μ values between nano and micro composites was 17.84%, achieved for the P-45nZ sample at 59.53 keV. These findings demonstrated that ZrO_2_ nanoparticles shield radiation more effectively than micro ZrO_2_ even at the same photon energy and filler wt%. Thus, the proposed nano ZrO_2_/PMMA composites can be used as effective shielding materials to lessen the transmitted radiation dose in radiation facilities.

## Introduction

In the current era, ionizing radiation has been employed worldwide in many fields, such as medical diagnostics and treatments^1^, nuclear facilities, agricultural and industrial applications, mining areas, and research^2^. In addition to these well-known fields, ionizing radiation is also used to screen people for non-medical reasons at border installations and military checkpoints, specifically to find bulk bombs or other illegal items concealed on the body, such as substances not picked up by metal detectors. Advanced imaging technology, such as accelerator-based container scanners and baggage inspection scanners with small, medium, and high energy, have been used in some countries for customs inspections against any threats that affect the countries’ border security. Some X-ray inspection systems, such as the Orion® 928DX inspection system, use X-rays with energies of nearly 168 keV in airports^3^. Furthermore, the Eagle® G60 ZBx gantry inspection system uses X-rays with dual-energy 3 and 6 MeV in ports^4^. The introduction of X-ray inspection systems at the airline’s checkpoints and border control was met with social controversy in part connected to exposure dose to travelling people, operators and bystanders. Ionizing radiations employed in such systems may provide health risks if improperly handled or the equipment’s safety features are deficient. Well-known negative consequences of exposure to ionizing radiation on bone health include a reduction in mineral density, bone growth retardation in children, and spontaneous fractures in older women^5^, and it primarily delivers harmful effects to damage DNA^6^.

To reduce the drawbacks of ionizing radiation, appropriate safety precautions must be implemented to balance their potential benefits against their drawbacks. International radiation groups have proposed specific guidelines to lessen and reduce the risks of radiation exposure to human organs. ALARA (As Low As Reasonably Achievable) is the most popular radiation protection rule that is based on reducing radiation doses by minimizing the time of radiation exposure, putting as much distance as possible between the user and the source of radiation, and the usage of radiation shields must be applied^7^. Lead and concrete are the traditional and common shielding materials utilized as protective materials in radiation facilities. Concrete is an excellent shielding material that has attracted significant interest because of its affordability, environmental friendliness, optimum density for radiation attenuation, easily shaped, requires little upkeep, and strong mechanical properties^8^. It is also widely used as an effective radiation shield for the X-ray inspection systems such as the EagleP60® ZBx and the Eagle M60® ZBx inspection systems located at the ports and border points of the country^9^. However, due to flaws like cracking and immobility, it is useless for other applications. Lead is the most commonly utilized radiation shielding material due to its high density, low cost, and superior shielding performance^10^. Even while lead seems like the perfect material for a shield, its toxicity^11^ toward people and the environment makes it less valuable. The need for lead alternatives has increased recently, particularly in the medical industry^12^.

Numerous materials’ characteristics have been created and enhanced for use as radiation shielding to overcome these drawbacks. The chemical stability, flexibility, lightweight, and low cost of polymer composites with inorganic fillers like micro and nanoparticles have led to extensive research into these materials as potential substitutes for conventional radiation shielding materials^13^. There are many polymers, including PMMA^14^, polyethylene^15^, polypropylene^16^, polyvinyl chloride^17^, epoxy^18^, styrene-butadiene rubber^19^, natural rubber^20^, silicon rubber^21^, ethylene-propylene-dine monomer (EPDM)^22^, polystyrene^23^, and recycled polymers^24,25^, were studied as radiation-protective matrixes. Nagaraja et al. investigated the performance of radiation shielding for different types of polymers that are commonly used within the energy range 81–1332 keV. Among all the tested polymers, the lead tetragonal polymer is the most effective γ-rays absorber^26^. The polymer matrix has been filled with metal oxides like PbO^27^, CdO^28^, Bi_2_O_3_^29^, ZnO^30^, Gd_2_O_3_^31^, and MgO^32^ to develop a radiation shield that can be used to attenuate X-rays and γ-rays.

Metal oxide fillers reinforced in polymer composites at the nanoscale can significantly improve their mechanical, electrical, and optical properties. Additionally, their small size makes these materials extremely effective at attenuating radiation. For example, El-Khatib et al. compared the radiation-shielding abilities of different loadings of micro and nano CdO distributed HDPE matrix at photon energy ranging from 59.53 to 1408.01 keV and reported that nano-CdO/HDPE composites shielded γ-rays more effectively than micro-CdO/HDPE composites at the same weight fraction^33^. Another research investigated the radiation shielding ability of epoxy-based micro and nano WO_3_ and Bi_2_O_3_ reinforced composites^34^. The study demonstrated that nano dopant is more successful and effective in attenuating the photons. Abbas et al. studied the effect of Bi_2_O_3_ micro- and nano-particles content on silicon rubber’s (SR) γ-ray interaction parameters^35^. The attenuation coefficients of the obtained SR samples showed a clear advantage in lower energy levels compared to other energies. Furthermore, the SR’s nano-Bi_2_O_3_ was superior to the SR’s micro-Bi_2_O_3_. Additionally, the mechanical results revealed that the material’s flexibility decreased as the Bi_2_O_3_ filler was increased to 30%.

Poly-methyl methacrylate (PMMA) is a significant kind of polymer among thermoplastics. PMMA is an optically transparent polymer with a refractive index of 1.49 and a density of 1.20 and is frequently used as an alternative to inorganic glass^36,37^. PMMA is an amorphous polymer that resists corrosion, abrasion, weather, and chemicals, as well as its ideal production conditions are lightweight and resistant to breaking. Numerous products, including coatings, additives, sealants, optical fibers, and neutron stoppers, have been made with PMMA. By incorporating filler into the PMMA matrix, this versatile material’s applications may be even more varied because well-dispersed filler could improve some of its physical characteristics^38^. Chen et al. evaluated the shielding properties of different samples, including pure PMMA, PMMA/MWCNT, and PMMA/MWCNT/Bi_2_O_3_, compared with aluminum (Al). According to the electron-beam attenuation properties, the PMMA/MWCNT/Bi_2_O_3_ nanocomposite was 37% lighter than Al while still providing the same level of radiation protection in the 9–20 MeV electron energy range^39^. Cao et al. investigated the performance of γ-rays shielding and the physical and mechanical characteristics of PMMA composites doped with 0–44.0 wt% Bi_2_O_3_ prepared by the fast-curing technique. The results showed that for radiation energies up to 1000 keV, PMMA/Bi_2_O_3_ composites showed superior γ-rays shielding performance compared to pure PMMA. Additionally, the hardness measurement shows that mechanical hardness rises with increasing loading of Bi_2_O_3_^40^. Another research reported the use of PMMA/Bi_2_O_3_ polymer composites as a replacement for concrete and gypsum in the construction of diagnostics radiation facilities^41^. Furthermore, lightweight, environment-friendly, and cost-effective materials based on flexible Bi-PMMA composites are investigated as radiation shielding materials suitable for low energy γ-rays^42^. Recently, PMMA polymer with different concentrations (0, 2, 5, 10, 15, and 20 wt%) of BaTiO_3_ as a nanofiller was examined to be used as nuclear radiation shielding materials. The developed non-toxic, flexible, and transparent nanocomposite protective material is presented and the specimens containing 10–15 wt%, showed enhanced radiation attenuation^43^.

Zirconium oxide (ZrO_2_), also known as Zirconia, is a material with significant technological importance due to its outstanding corrosion resistance, high strength, high chemical stability, chemical and microbiological resistance, and high mass attenuation coefficient^44^. Furthermore, due to its excellent thermal stability and low neutron absorption cross-section, Zirconia is applied in nuclear reactor technology. Wahab et al. evaluated the effect of zirconia nanoparticles on the radiation shielding performance of the lead borate glass^45^. Regarding the considerable collection of research indicated above, incorporating nanofillers into polymers is a promising method to develop novel radiation protective materials. There is also a strong need for more research to study how the filler size affects the shielding characteristics against γ-rays for various composite systems.

The survey of the literature reveals that there are very limited studies that deal with the use of nano ZrO_2_ as a filler in the polymeric matrix to attenuate γ-rays. Hence, the primary goal of this work is to investigate how the particle size and weight percentage of ZrO_2_ affect the ability of ZrO_2_/PMMA composites to shield against γ-rays. For this purpose, the mass attenuation coefficients of pure PMMA and PMMA loaded with 15, 30, and 45 wt% of micro and nano ZrO_2_ were estimated experimentally and compared to results obtained theoretically from XCOM database. Additionally, other shielding parameters such as the linear attenuation coefficients (μ), half-value layer (HVL), tenth value layer (TVL), mean free pass (MFP), effective atomic number (Z_eff_), effective electron density (N_eff_), and equivalent atomic number (Z_eq_), as well as exposure buildup factor (EBF) and energy absorption buildup factor (EABF) were calculated at various energies between 0.015 and 15 MeV to assess the γ-rays shielding ability of the prepared ZrO_2_/PMMA composites.

## Materials and methods

### Materials

Self-cured acrylic resin (Acrostone Cold Cure Acrylic Resin), a commercial product with a density of 1.18 g/cm^3^, comes in two bottles containing powder (Poly methyl methacrylate, prepolymer (–(–CH2¼C(CH3) COOCH3-)n-PMMA) and (MMA) monomer liquid hardener (CH2¼C (CH3) COOCH3, MMA), were the matrix materials employed in this work and provided by the Acrostone Dental & Medical Supplies Company in Cairo, Egypt. The physical properties of MMA liquid hardener and PMMA powder are summarized in Tables 1 and 2, respectively. Zirconium oxide micro- and nanoparticles were employed as fillers and obtained from Nanoshel business Wilmington, DE 19808, USA. Micro Zirconium oxide particles were provided with a purity of 99.9% and an average size of about 1–2 µm. In comparison, the Zirconium oxide nanoparticles were supplied with a purity of 99.9% and an average size of about 80 nm. The physical properties of Zirconium oxide microparticles (MPs) and nanoparticles (NPs) are listed in Table 3.

**Table 1:** The physical properties of MMA^46^.

Physical properties	Boiling point	Density	Vapor pressure	Molecular weight
Clear, colorless liquid of strong odor	100 °C	0.943 g/cm^3^	38 hPa at 20 °C	100 g/mol

**Table 2:** The physical properties of PMMA^46^.

Physical properties	Bead diameter	Density	Molecular weight
Fine powder, polymer beads, soluble in liquid polymer	1–120 µm	1.18 g/${{\text{cm}}}^{3}$	0.8 g/mol

**Table 3:** The physical properties of ZrO_2_ MPs and ZrO_2_ NPs.

Zirconium powder	Purity (%)	APS	Color	Density (g/cm^3^)
ZrO_2_ MPs	99.9	40–50 µm	White	5.68
ZrO_2_ NPs	99.9	80 nm	White	5.68

### Cold (self)-cured PMMA

In comparison to heat-cured PMMA, cold-cured PMMA possesses a different composition and polymerization technique, making it unnecessary to apply thermal energy. It is also known as chemically cured or auto-polymerized PMMA, indicating that the polymerization process initiates immediately after the powder and liquid components are combined. Consequently, no heat is required for the polymerization reaction to occur since the benzoyl peroxide initiator present in the pre-polymerized PMMA pellets can be chemically activated. The advantages of cold-cured PMMA over heat-cured PMMA are its superior adaptability and dimensional stability, resulting in reduced polymerization shrinkage^47^.

### Preparation of ZrO_2_/PMMA composites

This study utilized the self-curing method to fabricate pure PMMA, micro- ZrO_2_/PMMA, and nano- ZrO_2_/PMMA composites. Table 4 lists the sample codes, compositions, and densities of the produced composites. Three main groups of acrylic-resin specimens were fabricated; (a) the reference group (P-0Z) was prepared by blending self-cured PMMA powder and liquid MMA in a 3:1 by volume ratio as recommended by the manufacturer, (b) the modified micro ZrO_2_/PMMA group (P-15mZ, P-30mZ, P-45mZ) which was fabricated at constant micro filler loadings of about 15, 30 and 45 wt%, and (C) the modified nano-ZrO_2_/PMMA group (P-15nZ, P-30nZ, P-45nZ) which was fabricated with the same loadings of nanofiller.

**Table 4:** Sample codes, weight fractions, and densities of ZrO_2_/PMMA composites.

Sample codes	Composition (wt%)			Density (g/cm^3^)
	PMMA	ZrO_2_		
		Micro	Nano	
P-0Z	100	–	–	1.176 ± 0.007
P-15mZ	85	15	–	1.328 ± 0.006
P-15nZ	85	–	15	1.390 ± 0.005
P-30mZ	70	30	–	1.516 ± 0.004
P-30nZ	70	–	30	1.593 ± 0.009
P-45mZ	55	45	–	1.731 ± 0.005
P-45nZ	55	–	45	1.833 ± 0.008

**Figure 1:**
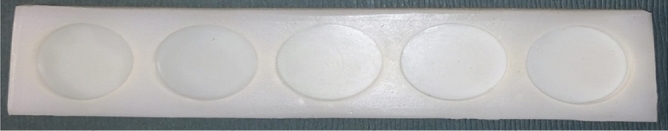
Silicon rubber mold.

**Figure 2:**
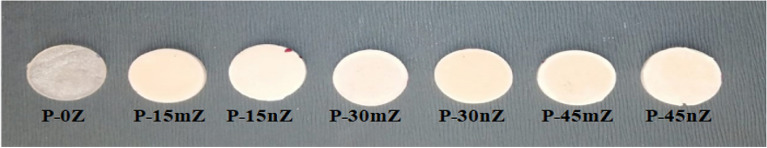
The prepared specimen’s final shape.

Before preparing the samples, each component was pre-weighed according to the weight fractions listed in Table 4, using a 0.0001 g sensitive electrical balance (Analytical Balance, GR200, Japan). The reference group (P-0Z) was prepared by mixing dry powder (PMMA) with liquid (MMA) in a clean and dry glass beaker and stirred continuously at room temperature for a maximum of 4.5 min (according to the manufacturer) to eliminate any gas bubbles from the specimens. The mixing process was performed using a mechanical mixer set to speed 20 rpm until the mixture reached the dough stage. Then, the mixture was poured into the center of an opening silicone rubber mold, shown in Fig. 1, until the mold was filled. The mold must be slowly shaken and pulsed from side to side. The mold was then kept standing on the workbench at room temperature for 20 min after the mixing process had started to allow the mixture to thicken and harden the surface of the casting. The modified groups were prepared by mixing ZrO_2_ nanoparticles or ZrO_2_ microparticles with the PMMA powder in a glass beaker and mixed with an electric mixer for 20 min to create a homogenous mixture. Then, as previously mentioned, the blended powder was added as one unit to the liquid monomer in a ratio of 3:1 by volume. When the mixed acrylic resin reached the dough stage, it was packed in a silicon rubber mold for 2 h at room temperature (25 ± 2 °C) to get the specimen’s final shape, as shown in Fig. 2.

### Morphology study and structural characterization

The particle size of micro and nano ZrO_2_ powder was analyzed using a transmission electron microscope (TEM) (JEM 1400 Plus, JEOL, Japan) at 200 kV. Furthermore, the cross-section morphologies of the prepared samples were examined using a scanning electron microscope (SEM) (JSM-6010LV, JEOL). To enhance the SEM image quality, a thin layer of gold (20 nm) was coated on the prepared samples using a low-vacuum sputtered coating system (JEOL-JFC-1100E). SEM images were acquired to compare the dispersion of ZrO_2_ micro and nanoparticles within the PMMA matrix. TEM and SEM analyses were performed to study how the ZrO_2_ particle size and its distribution affected the shielding properties of the investigated ZrO_2_/PMMA composites. The present specimens were also analyzed using a Bruker Vertex 70 infrared spectrometer in the 4000–400 range, together with a Platinum ATR unit, Germany-Ray diffractometer (Schimadzu-7000), to collect FT-IR data and examine photon absorption and transmission in the IR region. FT-IR analysis was conducted to identify the functional groups in the ZrO_2_/PMMA composites and the interaction mechanism of ZrO_2_ particles and PMMA polymeric matrix.

### γ-ray spectroscopic setup

**Figure 3:**
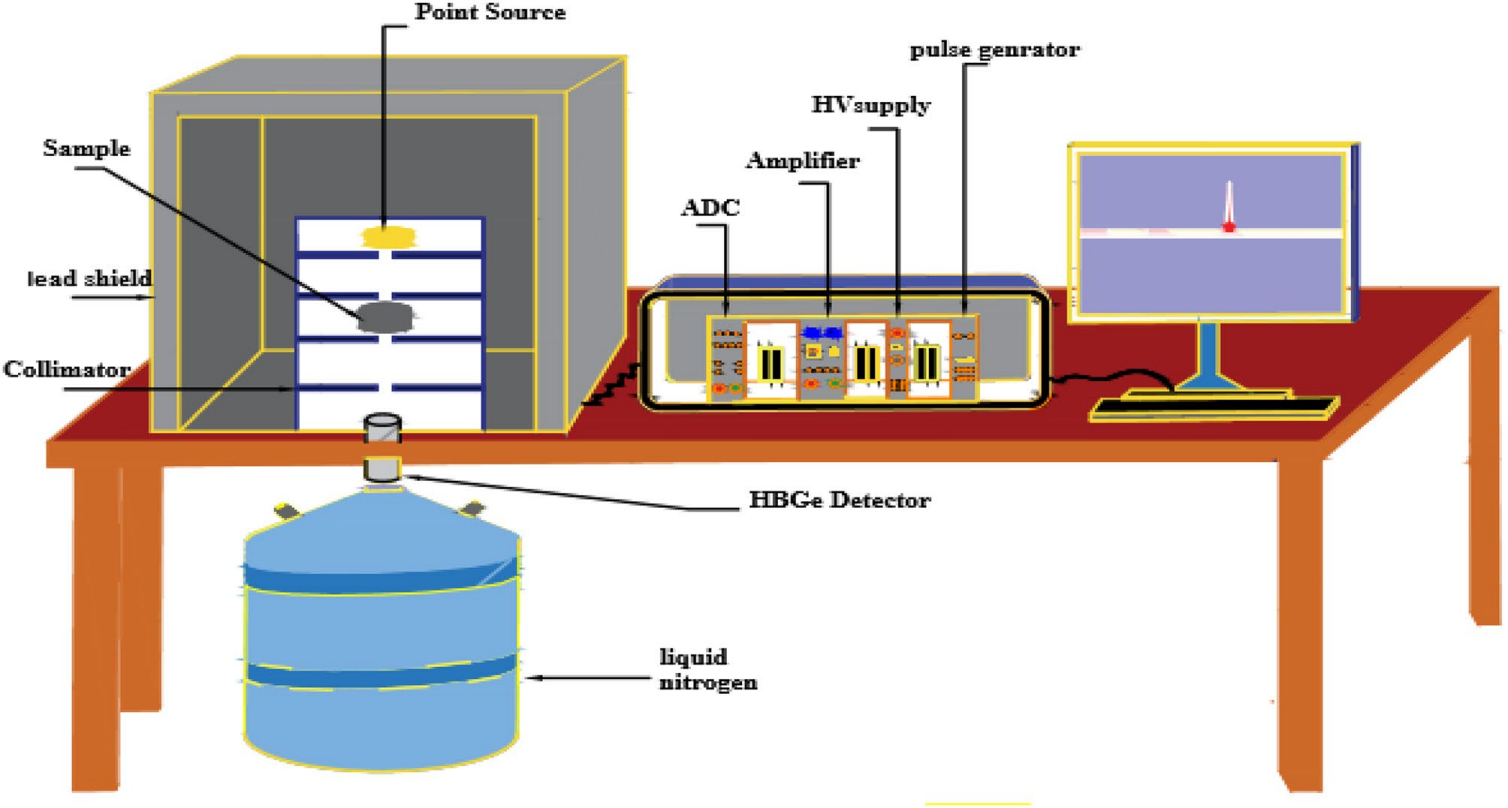
Experimental arrangement for the γ-rays measuring system.

The cylindrical high purity germanium detector (Model GC1520 from Canberra, United States) was employed in conjunction with a multichannel analyzer to conduct γ-ray spectroscopic measurements. The detector boasts a relative efficiency of 15% within the 50 keV to 10 MeV range, with a resolution of 1.85 keV at the 1.33 MeV γ-ray peak using Co-60^48^. The detector was encased with a 15 cm thick lead shield to mitigate background radiation. The measurements were calibrated using standardized sources, including Am-241, Ba-133, Cs-137, Co-60, and Eu-152, in the energy range between 0.05953 and 1.4081 MeV. Table 5 outlines the emitted energies and activities attributed to these sources. The experimental arrangement for the γ-ray measuring system is illustrated in Fig. 3.

**Table 5:** Standard radioactive point sources and their emitted photon energies and activities.

Radioactive sources	Photon energy (MeV)	Activity (kBq)
Am-241	0.05953	253.250
Ba-133	0.08099	109.598
	0.35601	
Cs-137	0.66166	278.997
Co-60	1.17323	33.658
	1.3325	
Eu-152	0.12178	141.590
	0.24469	
	0.34428	
	0.7789	
	0.96413	
	1.4081

**Figure 4:**
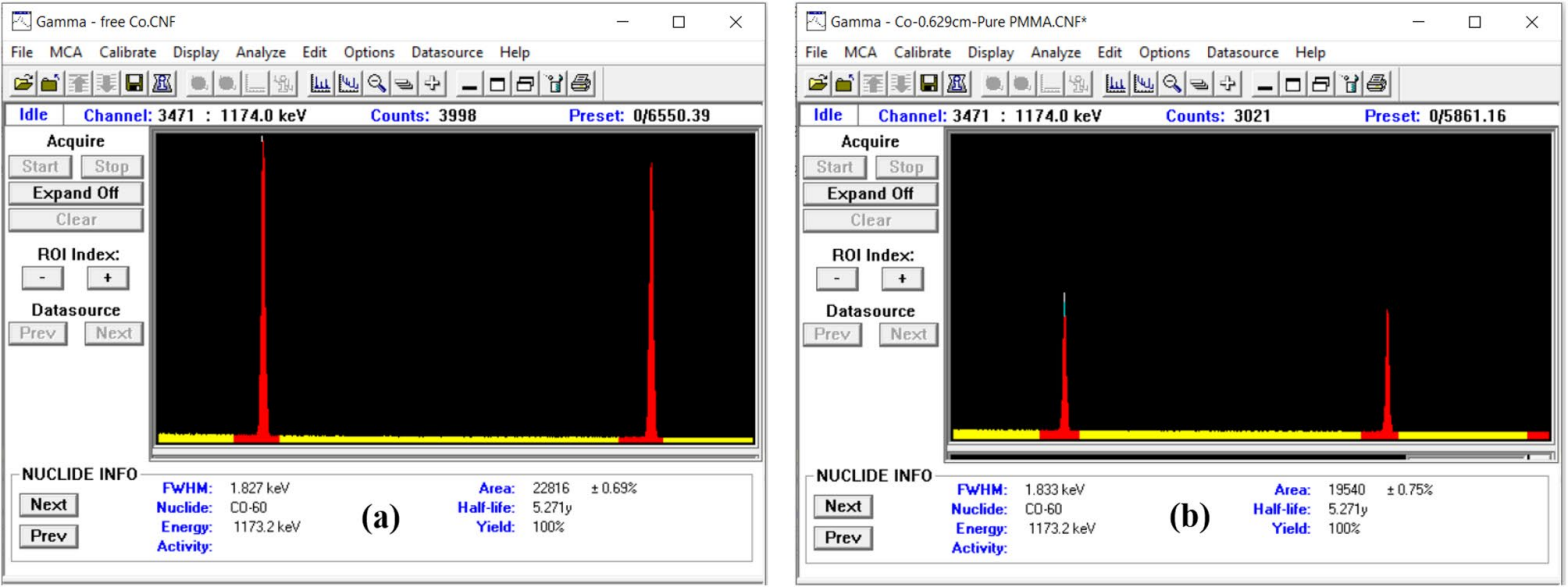
The obtained spectra using Co-60 radioactive source: (**a**) without absorber and (**b**) with P-0Z sample.

The γ-ray spectrum for each measurement was obtained based on the sample thickness, such that the statistical error was less than 1%. For example, Fig. 4 depicts the obtained spectra using a Co-60 radioactive point source in the absence and presence of the P-0Z sample. The electrical signal generated by the detector was amplified and analyzed using Canberra’s Genie 2000 data acquisition and analysis software ISO 9001. The net area beneath the photo peak was calculated and divided by the acquisition time to determine the count rate. The counting rate was determined in the presence (I) and absence (I_0_) of the specimen, respectively. Beer–Lambert’s law (Eq. 1) was then utilized to determine the linear attenuation coefficient (cm^−1^) of each sample at various γ-ray energies.

### Attenuation parameters calculations

The linear attenuation coefficient (μ), a significant shielding parameter for determining the impact of γ-rays at proper energy on the materials under study, can be calculated from Beer–Lambert’s law as indicated in Eq. (1)^49^:

1 $$\mu =1/t \,{\text{ln}}\frac{{I}_{0}}{I},$$

where the initial (I_0_) and transmitted (I$$)$$ intensities across the sample with a thickness (t) can be experimentally calculated as discussed in “γ-ray spectroscopic setup” section. The MAC can be derived using Eq. (2):

2 $$MAC=\frac{\mu }{{\rho }_{s} },$$

where ρ_s_ is the density of the sample measured using Archimedes method^50^:

3 $${\rho }_{s}=\frac{({m}_{1})}{\left({m}_{1}-{m}_{2}\right)}\cdot {\rho }_{1},$$

where ρ_s_ refers to the density of the samples, ${\rho }_{1}$ is the density of water at the test temperature, ${m}_{1}$ is the mass of the dry sample, and m_2_ is the mass of the immersion sample.

In developing an appropriate radiation shielding substance, two factors must be considered: the HVL and TVL. HVL and TVL are the material thicknesses required to reduce the γ-ray intensity to 50% and 10% of its original value, respectively. Equations (4) and (5) are typically used to estimate these values^51^:

4 $$HVL=\frac{{\text{ln}}2}{\mu },$$

5 $$TVL=\frac{{\text{ln}}10}{\mu }.$$

The MFP, which is defined as the average distance moved by a photon between two subsequent reactions, this parameter was given in Eq. (6)^52^:

6 $$MFP=\frac{1}{\mu }.$$

To determine a material’s radiation shielding capabilities, it is necessary to calculate its Z_eff_ and N_eff_ parameters. These values are obtained from the atomic cross section (σ_a_) and electron cross section (σ_e_) of the material. The σ_a_ and σ_e_ values directly relate to the number of atoms and electrons present in a unit volume of the material. Materials with higher σ_a_ and σ_e_ values are more effective as shielding materials. σ_a_ is calculated using Eq. (7)^53^ and provides the probability of interaction per atom within the material’s unit volume.

7 $${\sigma }_{a}=\frac{( \frac{\mu }{\rho } )}{N{\Sigma }_{i}\frac{{w}_{i}}{{A}_{i}}},$$

where N is Avogadro’s number, A_i_ and f_i_ are the atomic weight and fractional weight for each target element, respectively. σ_e_ provides the probability of interaction per electron in the specimen’s unit volume and is expressed by Eq. (8)^54^:

8 $${\sigma }_{e}= \frac{1}{N}{\Sigma }_{i}\left(\frac{\mu }{\rho }\right)\frac{{f}_{i}{A}_{i}}{{z}_{i}},$$

where Z_i_ is the atomic number and f_i_ is the target element’s fractional abundance. Along with the use of σ_a_ and, σ_e_. The effective atomic number is derived by applying Eq. (9)^55^:

9 $${Z}_{eff}=\frac{{\sigma }_{a}}{{\sigma }_{e}}.$$

The N_eff_ values are referred to the electron numbers per unit mass of the interacting target and is given by Eq. (10)^56^:

10 $${{\text{N}}}_{eff}=N\frac{{{\text{Z}}}_{eff}}{{{\Sigma }_{i}f}_{i}{A}_{i}}.$$

When constructing a shielding material, the two forms of buildup factor, the EABF and the EBF are crucial parameters that should be taken into account. Due to secondary γ-rays emissions^57^, buildup factors are always more than one and correct the attenuation estimations in Beer–Lambert’s law. The three stages below were followed using the Geometric Progression (GP) fitting technique to compute the EABF and EBF for the produced ZrO_2_/PMMA composites:

(i) the composite’s Z_eq_ values, which is identical to the elemental atomic number, was initially calculated with the following Eq. (11)^58^:
  11 $${Z}_{eq}=\frac{{Z}_{1}\left({\text{log}}{R}_{2}-{\text{log}}R\right)+{Z}_{2}({\text{log}}R-{\text{log}}{R}_{1})}{({\text{log}}{R}_{2}-{\text{log}}{R}_{1})},$$
  where R_1_ and R_2_ represent the (μ_Comp_/μ_total_) ratios for the elements with atomic numbers Z_1_ and Z_2_, and R represents the (μ_Comp_/μ_total_) ratio for the composite under investigation at a particular energy that falls between ratios R_1_ and R_2_.
(ii) After getting the Z_eq_ values of the specified composites were then employed to estimate the GP fitting EABF and EBF coefficients [b, c, a, X_k_, and d] in the range of energies (0.015–15 MeV) using the following formula^59^:
  12 $$C=\frac{{c}_{1}\left({\text{log}}{Z}_{2}-{\text{log}}{Z}_{eq}\right)+{c}_{2}\left({\text{log}}{Z}_{eq}-{\text{log}}{Z}_{1}\right)}{({\text{log}}{Z}_{2}-{\text{log}}{Z}_{1})}.$$
  C_1_ and C_2_ are the GP fitting parameters obtained from ANSI/ANS-6.4.3 standard data^60^, equivalent to the atomic numbers Z_1_ and Z_2_ at which Z_eq_ of the produced ZrO_2_/PMMA composites are located.
(iii) Eventually, the resulting GP fitting parameters were used to compute the EABF and EBF using the following relationships^61^:
  13 $$B\left(E,x\right) =\left[ 1+\frac{b-1}{k-1}\left({k}^{x}-1\right)\right], k\ne 1,$$
  and
  14 $$B\left(E,x\right) =\left[1+\left( b-1\right)x\right] , k=1,$$
  where
  15 $$k\left(E,x\right) =\left[c{x}^{a}+d\frac{{\text{tan}}h\left(x/ {X}_{k}-2\right )-{\text{tan}}h\left(-2\right)}{1-{\text{tan}}h\left(-2\right)}\right]\, for \,x\le 40 \, {\text{mfp}},$$
  where *x* represents the penetration depth in terms of MFP and E represents the energy of the incident photon.

## Result and discussion

### Characterization

#### Transmission electron microscope (TEM) analysis

**Figure 5:**
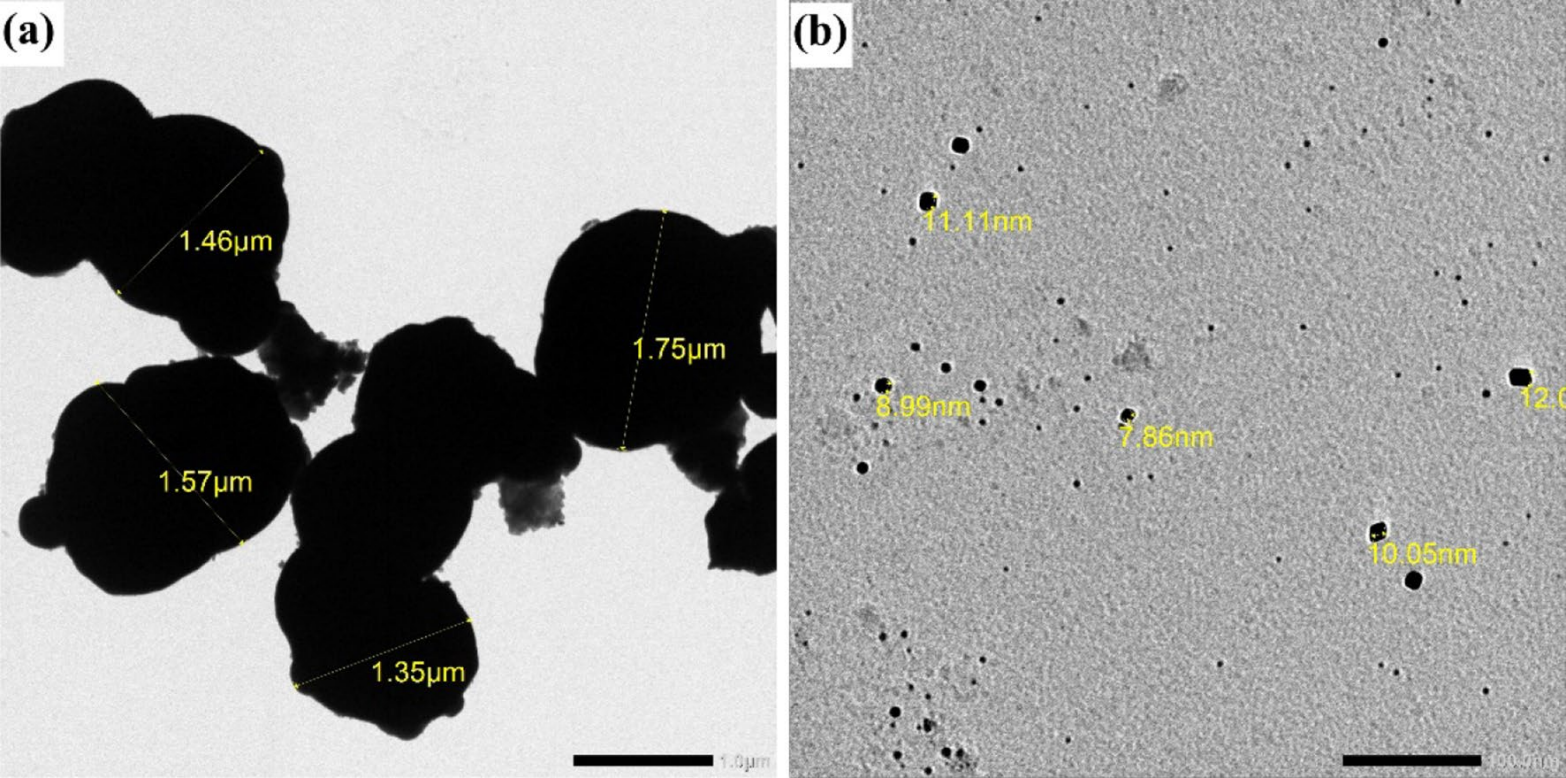
TEM images of (**a**) ZrO_2_ MPs and (**b**) ZrO_2_ NPs.

The TEM micrographs of ZrO_2_ MPs and NPs are depicted in Fig. 5. Figure 5a shows that ZrO_2_ MPs are nearly spherical in shape and have an average particle size between 1.46 and 1.75 µm. On the other hand, Fig. 5b demonstrates the existence of ZrO_2_ NPs, which have a consistent size distribution between 7.86 and 12 nm.

#### Scanning electron microscope (SEM) analysis

**Figure 6:**
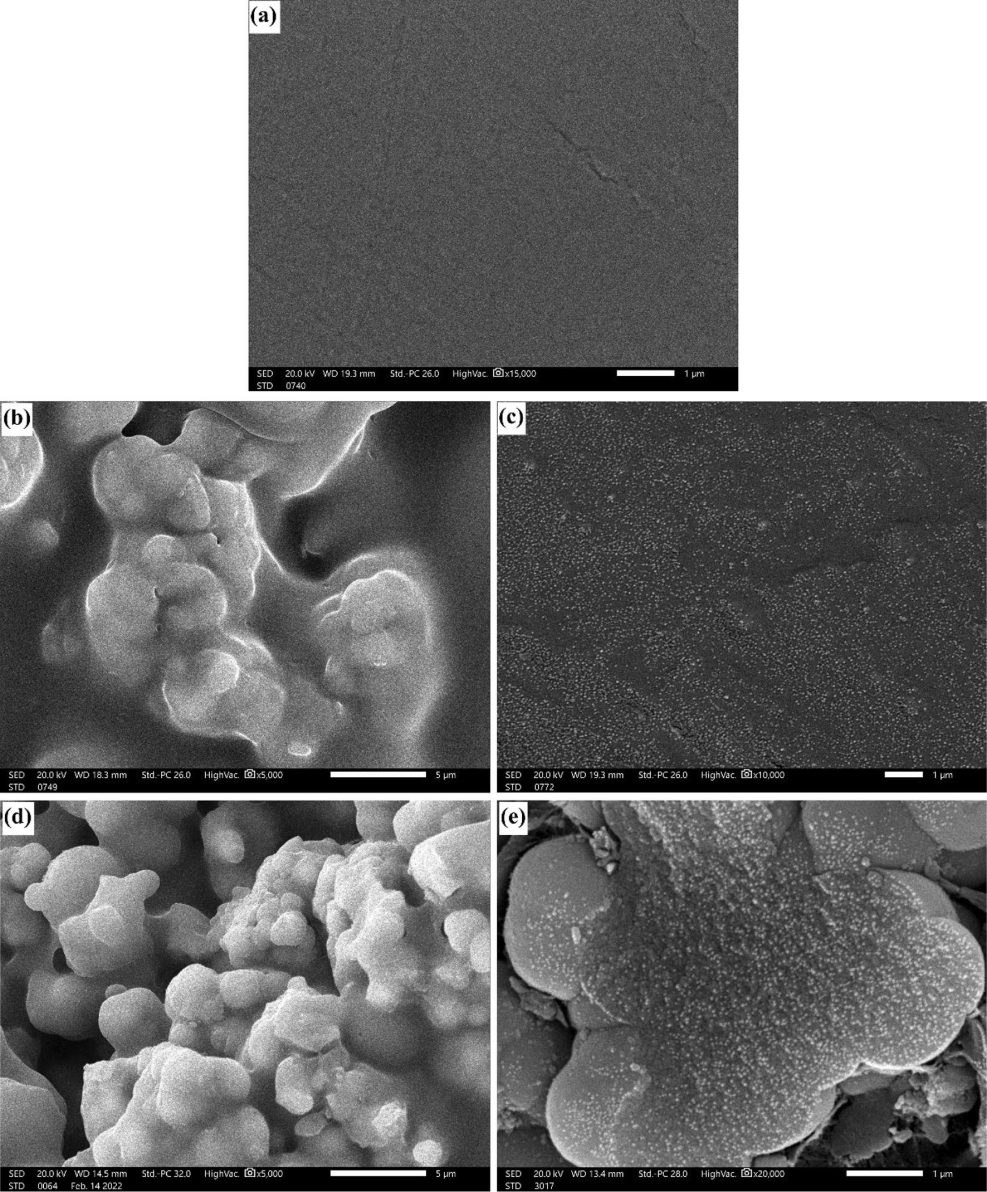
SEM images of (**a**) P-0Z, (**b**) P-15mZ, (**c**) P-15nZ, (**d**) P-45mZ, and (**e**) P-45nZ composites.

Figure 6 displays the SEM images of P-0Z, P-15mZ, P-15nZ, P-45mZ, and P-45nZ composites. Figure 6 describes how ZrO_2_ particles impacted PMMA on a micro and nanoscale. Figure 6a depicts smooth and distinct variation compared to ZrO_2_/PMMA composites. In other words, pure PMMA (Fig. 6a) and ZrO_2_/PMMA composites (Fig. 6b–e) exhibit a distinct difference in morphology. The SEM images of micro and nano ZrO_2_/PMMA composite samples with identical filler wt% are compared as indicated in Fig. 6b–d. It is clear that ZrO_2_ Nps are uniformly scattered and thoroughly incorporated into the PMMA matrix in ZrO_2_/PMMA composites, which may strengthen the interfacial adhesion between the PMMA matrix and ZrO_2_ NPs and offer an interconnecting structure for shielding. While large ZrO_2_ MPs are not fully covered by the PMMA matrix in micro ZrO_2_/PMMA composites, and some of them peel off from the matrix due to insufficient interfacial adhesion, which behaves as voids for shielding. Additionally, it was evident that the porosity reduces and the distribution rises as the fraction of particles increases. This attribute proves that increasing the ZrO_2_ content in PMMA will enhance structural, mechanical and shielding properties.

#### Fourier transform-infrared (FT-IR)

**Figure 7:**
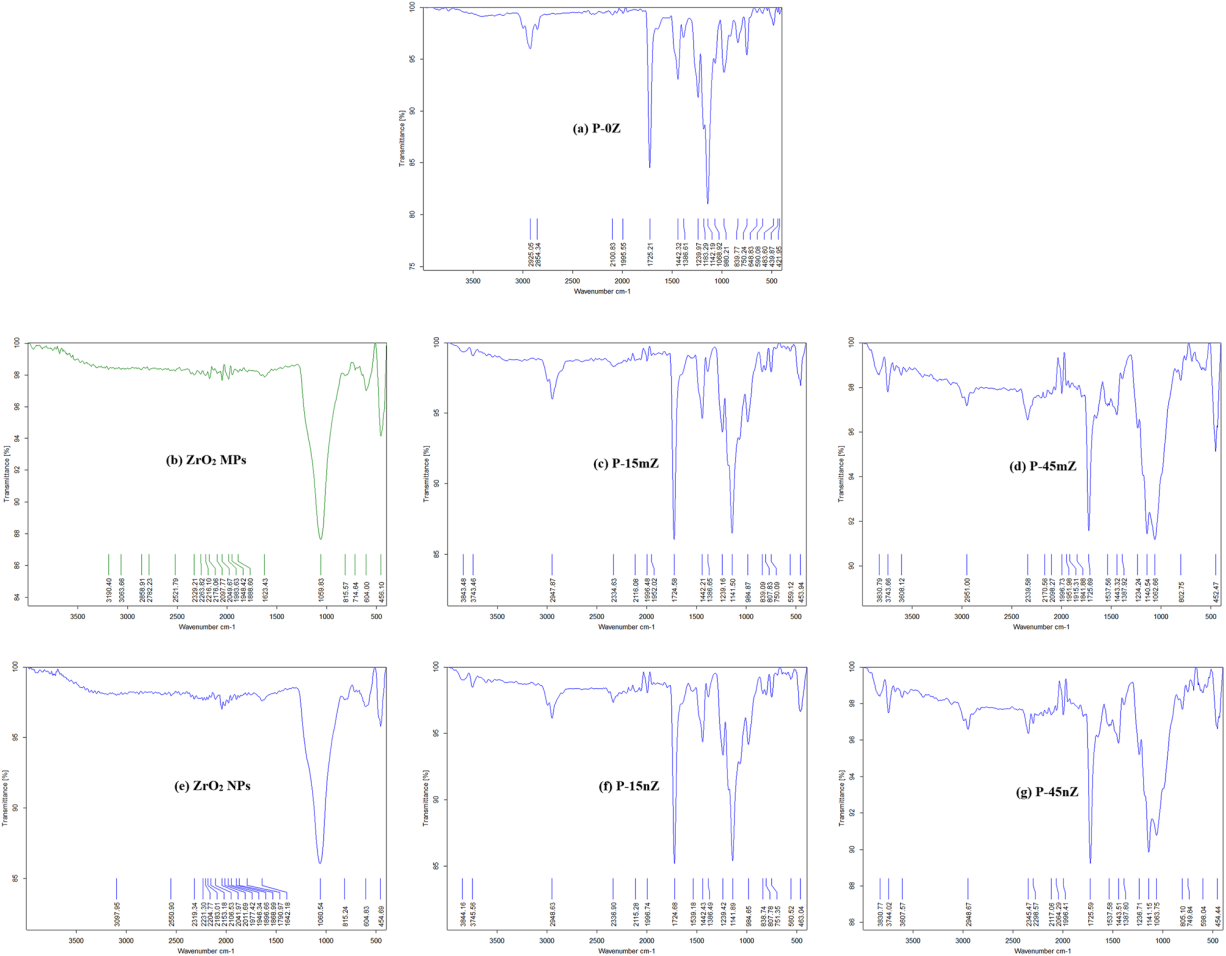
FT-IR spectrum of (**a**) P-0Z, (**b**) ZrO_2_ MPs, (**c**) P-15mZ, (**d**) P-45mZ, (**e**) ZrO_2_ NPs, (**f**) P-15nZ, and (**g**) P-45nZ samples.

FT-IR spectroscopic analysis was performed on the produced samples using Bruker Vertex 70 infrared spectrometer to identify the functional groups in the ZrO_2_/PMMA composites and the interaction mechanism of ZrO_2_ particles and PMMA polymeric matrix. The FT-IR spectra of P-0Z, ZrO_2_ MPs, P-15mZ, P-45mZ, ZrO_2_ NPs, P-15nZ, and P-45nZ samples were collected in the wavelength region of the 400–4000 cm^−1^ and displayed in Fig. 7. A discrete absorption band from 1142.19 to 1239 cm^−1^ can be seen in the FT-IR spectrum P-0Z as depicted in Fig. 7a, which related to the C–O–C stretching vibration. The vibrations of the –methyl group can be identified to the pair of bands at 1386 cm^−1^, and 750 cm^−1^. The band at 980 cm^−1^ is the characteristic absorption vibrations of PMMA, jointly with the bands at 1068 cm^−1^ and 839 cm^−1^. Due to the existence of ester carbonyl group stretching vibration (Acrylate carboxyl group), a sharp intensity peak at 1725.21 cm^−1^ was conducted. The band at 1442.32 cm^−1^ can be attributed to the bending vibration of the C–H bonds of the –CH_3_ group. The band at 1442.32 cm^−1^ can be allocated to the bending vibration of the C–H bonds of the –CH_3_ group. The two bands at 2925.05 cm^−1^ and 2854.34 cm^−1^ can be linked to the C–H bond stretching vibrations of the –CH_3_ and –CH_2_– groups, respectively. In addition, two faint absorption bands at 3734 cm^−1^ and 1644 cm^−1^ result from the stretching and bending vibrations of the –OH group, respectively^62^.

A broad peak can be seen in the FT-IR spectrum of ZrO_2_ MPs in Fig. 7b at 3428.96 cm^−1^, 1634.05 cm^−1^, and (456.10, 604 cm^−1^), which correspond to OH stretching, OH bending, and the Zr–O band, respectively. Peak locations, forms, and intensities have been determined in accordance with the fingerprint features, together with the material’s fundamental components^63^. As seen in Fig. 7c, the spectrum of the P-15mZ composite, compared to that of P-0Z, had new peaks at 453.94 cm^−1^ and 599.12 cm^−1^ referring to the metal–oxygen bond in ZrO_2_. Furthermore, it is clear that when the weight fraction of ZrO_2_ MPs was increased to 45 wt%, two peaks, as shown in Fig. 7d, were seen at 631 cm^−1^ and 698 cm^−1^, which are associated with stretching vibration modes of Zr–O and bending vibration of =C–H^64^. The apparent presence of these peaks indicates that ZrO_2_ MPs have been successfully embedded in the PMMA polymeric matrix.

Figure 7e displays the FT-IR spectrum of ZrO_2_ NPs. The peaks at 453.92 cm^−1^ and 599.12 cm^−1^ are related to the strong metal–oxygen conjunction in ZrO_2_ NPs, and two peaks at 631 cm^−1^ and 698 cm^−1^ are attributed to the Zr–O bond and bending vibration of =C–H as previously mentioned. At 574 cm^−1^ and 708 cm^−1^, two additional bands have been observed that are connected to the ZrO_2_ NPs. It is also clear from Fig. 7f,g that the spectra of P-15nZ and P-45nZ composites exhibit behavior that is comparable to that previously reported, in conjunction with the existence of two more peaks at 551 cm^−1^ and 598.04 cm^−1^ that were formed at greater concentrations of ZrO_2_ NPs. Consequently, it can be deduced from Fig. 7 that there was a chemical bond between the PMMA matrix and the ZrO_2_ filler in all composites, which resulted in chemical interactions between them.

### γ-ray shielding results

The MAC is a standard parameter utilized for measuring and comparing the performance of various shielding materials. Table 6 displays the experimentally measured values of MACs for all the investigated composites (pure PMMA, micro ZrO_2_/PMMA, and nano ZrO_2_/PMMA composites) in the energy range between 0.05953 and 1.4081 MeV. Furthermore, using the XCOM software, MAC for the PMMA and micro ZrO_2_/PMMA composites were generated theoretically, and the relative deviation (Δ%) between the experimental and theoretical results that were determined by using Eq. (16):

**Table 6:** Measured and theoretical values of MACs and their relative deviation (Δ%) for pure PMMA, micro ZrO_2_/PMMA and nano ZrO_2_/PMMA composites.

Sample	Photon energy (keV)	MAC (cm^2^ g^−1^)			
		Nano	Micro	XCOM	Δ%
Pure PMMA	0.05953		0.18248	0.1819	0.32
	0.08099		0.16879	0.1682	0.35
	0.12178		0.15298	0.1522	0.51
	0.24469		0.12276	0.1233	− 0.44
	0.35601		0.10731	0.1076	− 0.27
	0.66166		0.08401	0.0832	0.94
	0.7789		0.07679	0.0773	− 0.65
	0.96413		0.06956	0.0699	− 0.45
	1.17323		0.06395	0.0634	0.81
	1.3325		0.05927	0.0594	− 0.25
	1.4081		0.05825	0.0578	0.85
15 wt% ZrO_2_/PMMA	0.05953	0.61951	0.56627	0.5667	− 0.08
	0.08099	0.34900	0.32229	0.3227	− 0.13
	0.12178	0.21015	0.19578	0.1957	0.04
	0.24469	0.13533	0.12726	0.1262	0.84
	0.35601	0.11382	0.10768	0.107	0.64
	0.66166	0.08489	0.08133	0.08165	− 0.40
	0.7789	0.07950	0.07605	0.07574	0.41
	0.96413	0.07051	0.06852	0.06839	0.20
	1.17323	0.06331	0.06250	0.06205	0.73
	1.3325	0.05828	0.05798	0.05813	−0.25
	1.4081	0.05662	0.05648	0.05651	− 0.06
30 wt% ZrO_2_/PMMA	0.05953	1.03767	0.94591	0.9514	−0.58
	0.08099	0.52244	0.47889	0.4771	0.38
	0.12178	0.26084	0.24077	0.2393	0.61
	0.24469	0.13943	0.12995	0.1292	0.58
	0.35601	0.11175	0.10554	0.1065	−0.90
	0.66166	0.08412	0.08047	0.08008	0.49
	0.7789	0.07646	0.07388	0.07418	− 0.41
	0.96413	0.06906	0.06728	0.06691	0.56
	1.17323	0.06165	0.06069	0.06067	0.03
	1.3325	0.05738	0.05673	0.05684	−0.20
	1.4081	0.05481	0.05475	0.05527	−0.94
45 wt% ZrO_2_/PMMA	0.05953	1.44818	1.32409	1.336	−0.89
	0.08099	0.68241	0.62912	0.6315	−0.38
	0.12178	0.30566	0.28365	0.2829	0.27
	0.24469	0.13988	0.13114	0.1322	−0.80
	0.35601	0.11304	0.10630	0.1059	0.37
	0.66166	0.08303	0.07915	0.07851	0.81
	0.7789	0.07605	0.07279	0.07262	0.23
	0.96413	0.06754	0.06528	0.06543	−0.23
	1.17323	0.06105	0.05950	0.05928	0.38
	1.3325	0.05624	0.05546	0.05556	−0.18
	1.4081	0.05406	0.05373	0.05402	−0.54

16 $$\Delta \%=\left[{MAC}_{exp.} - {MAC}_{XCOM}\right)/{MAC}_{XCOM}]\times 100.$$

As can be seen from Table 6, both the MAC values obtained from XCOM and those achieved by laboratory measurement exhibit good comparability. This remark is accurate for all of the energies tested. However, some minor discrepancies were discovered between the two methods. These are acceptable because ordinarily, anyone can find a few minor errors in the experimental results, but generally, the experimental results are acceptable and agree with the XCOM results. This is a crucial and significant step since it clarifies the precision of the geometry used in the lab to calculate the MAC for the PMMA and ZrO_2_/PMMA composites. According to Table 6, the Δ% for pure PMMA (free of ZrO_2_ filler) is restricted to − 0.65 and 0.94%, whereas the Δ% for 15 wt% ZrO_2_/PMMA is restricted to − 0.40 and 0.84%, and for 30 wt% ZrO_2_/PMMA ranges between − 0.94 and 0.61%. However, it can only be between − 0.89 and 0.81% for 45 wt% ZrO_2_/PMMA. These results support that the practical and theoretical results are compatible since the Δ% is less than 2%.

**Figure 8:**
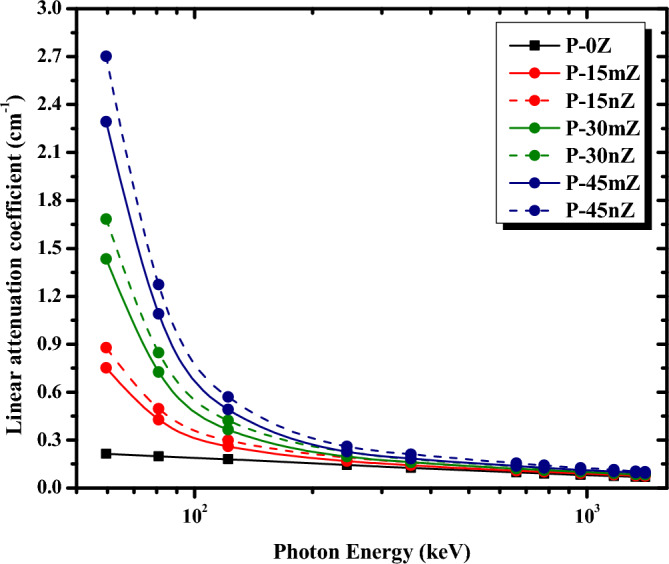
Comparison between linear attenuation coefficients of micro- and nano-ZrO_2_/PMMA composites at different ZrO_2_ concentrations as a function of photon energy.

The experimental results of μ values of pure PMMA, micro, and nano ZrO_2_/PMMA composites filled with various concentrations (15 wt%, 30 wt%, and 45 wt%) as a function of γ-ray energies are shown in Fig. 8. As can be seen from Fig. 8, the energy of the incident photons and the compositions of the protective material have a significant impact on μ values. The μ values have been found to significantly increase with increasing micro- and nano-ZrO_2_ concentrations in the composites and rapidly decline as photon energy increases. This trend may be demonstrated by focusing on the three primary processes by which energetic photons interact with matter; photoelectric effect, Compton scattering, and pair production, which all contribute to the energy loss of the incident photon. At energies less than 125 keV, the photoelectric effect is the primary mechanism that causes photons to be absorbed since the possibility of photoelectric absorption depends on Z^3^, where Z is the atomic number^65^. Therefore, due to element Zr with atomic number (Z = 40), μ values increase as the concentration of ZrO_2_ in the PMMA matrix increases. However, as photon energy increases beyond 125 keV, the likelihood of the photoelectric effect reduces roughly according to 1/E^3^^66^, where E is the energy of the incident photons, which illustrates why μ for every composite decreases slightly as the photon energy goes above 125 keV. Meanwhile, in this energy range increasing the ZrO_2_ filler wt% in the PMMA matrix is slightly affect the values of μ which have nearly the same value as the photon energy increase. This result is because, at this intermediate energy range, the effect of photoelectric absorption diminishes, and the Compton scattering mechanism takes over. Besides, the cross-section of the Compton scattering is practically independent of atomic number but depends on the number of electrons per unit mass.

Additionally, the difference of μ between micro- and nano-sized ZrO_2_/PMMA was also compared, as shown in Fig. 8. At all of the tested γ-ray energies, the nano ZrO_2_/PMMA curves always are above the micro ZrO_2_/PMMA curves for the same weight percent of ZrO_2_ filler. As the size of ZrO_2_ particles decreases from micro to nano size, the uniform distribution of ZrO_2_ NPs over a greater surface area within the PMMA matrix would increase the likelihood of incident photons interacting with ZrO_2_ NPs in nanocomposites as compared to micro composites and increase the probability of further scattering mechanisms for the photons until the photon’s energy is below 200 keV. Consequently, in PMMA-based radiation protective material, ZrO_2_ NPs exhibit excellent attenuation performance than ZrO_2_ MPs for the same chemical composition and weight percentage of the composite.

**Figure 9:**
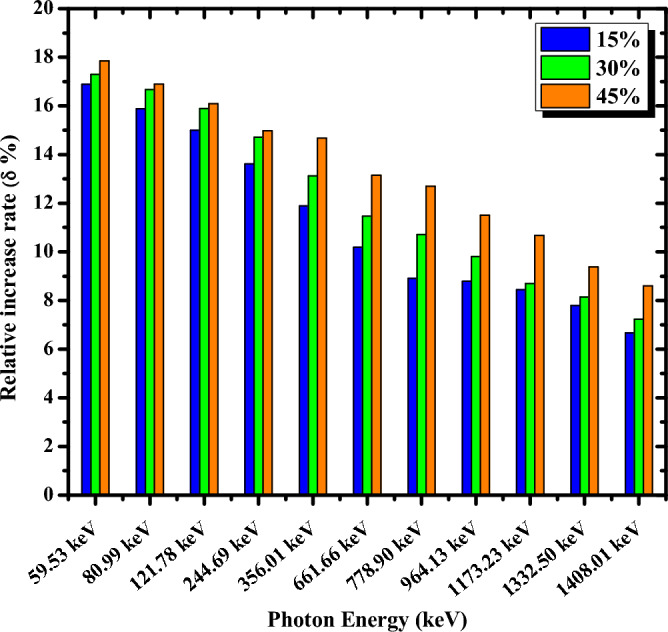
Relative increase rates (δ%) in relation to photon energy at various ZrO_2_weight percentages.

To evaluate the superiority of the shielding ability of nanocomposites over micro composites, the relative increase rate (δ%) in μ values between nano and micro ZrO_2_/PMMA composites was calculated according to Eq. (17) and depicted in Fig. 9 as a function of energy at various ZrO_2_ loadings.

17 $$\delta \%=[({\mu }_{\mathrm{nano }}- {\mu }_{{\text{micro}}}/{\mu }_{{\text{micro}}}]\times 100.$$

As shown in Fig. 9, the relative increase rate (δ%) increases with an increase in ZrO_2_ content; however, its value decreases as the photon’s energy increases from 0.05953 to 1.408 MeV. These findings suggest that, due to various photon interaction cross-sections at different photon energies, the size effect diminishes as photon energy increases. The absorption capability is Z-dependent when the photoelectric effect dominates at low photon energies. Due to the mid-high Z of the element zirconium in ZrO_2_ particles and the low Z of the elements C, O, and H in the PMMA matrix, the photoelectric absorption of ZrO_2_ particles is considerably higher than that of the PMMA matrix. As a result, these particles are extremely important for radiation shielding. At the energy of 0.05953 MeV, the δ% in the P-15nZ sample was 16.88%, compared to 17.29% in the P-30nZ sample and 17.84% in the P-45nZ sample. Since Compton scattering is more likely at higher energies and its cross-section can be regarded as the predominant interaction that does not rely on Z but rather on the free electrons, there is little difference in the ability of ZrO_2_ particles compared to the PMMA matrix. As a result, the essential role of ZrO_2_ particles diminishes, and the impact of particle size decreases. At the highest examined energy (1.408 MeV), the δ % was 6.67% for P-15nZ, whereas it was 7.23%and 8.60% for P-30nZ and P-45nZ samples, respectively. In summary, the δ % follows the general trend P-45nZ > P-30nZ > P-15nZ for all incident energies. So, the composite P-45nZ has superior shielding potentials over all the investigated samples.

**Figure 10:**
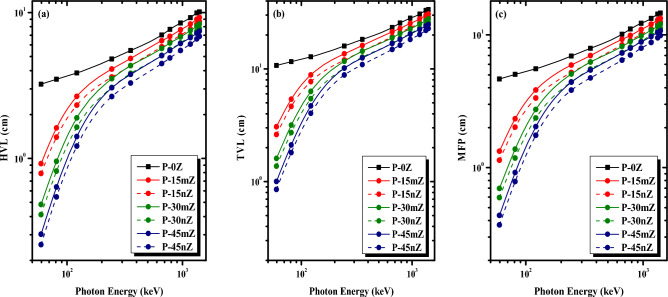
The variance of the (**a**) half value layer, (**b**) tenth value layer, (**c**) as well as mean free path, (**d**) with respect to energy for the studied polymers.

Three primary critical radiation shielding parameters, the HVL, the TVL, and the MFP have been researched in correlation to the radiation shielding capabilities of micro- and nano-structured composites^67^. Figure 10 displays the HVL, TVL, and MFP at different energies ranging from 0.0595 MeV to 1.408 MeV. The HVL is the thinnest sample at which 50% of the original γ-ray intensity passes through it. The findings of our calculation of the HVL for the chosen composites at the energies employed for the μ data are displayed in Fig. 10a. When analyzing the data in this Fig. 10a. One can see a gradual increase in HVL as energy is increased from 0.0595 to 1.408 MeV. This tendency indicates that the photons’ ability to penetrate samples rises along with their energy. The lowest HVL is found at 0.0595 MeV (in the range of 0.26111–3.2299 cm), and there is a significant increase across the upward energies (6.9944–10.1189 cm at 1.408 MeV), as shown in Fig. 10a. This fact emphasizes that as the radiation’s energy rises, more photons will be able to pass through the chosen samples. Figure 10a even further illustrates that the effective method for reducing the HVL and improving the ability of the chosen samples to attenuate γ-ray is the addition of ZrO_2_ to the PMMA matrix. Comparing P-45nZ to the other materials, P-45nZ presents the lowest HVL at any energy. Our analysis shows that the HVL appears in the following sequence: P-0Z > P-15mZ > P-15nZ > P-30mZ > P-30nZ > P-45mZ > P-45nZ. This pattern emphasizes that the addition of more ZrO_2_ improves photon shielding properties because ZrO_2_ is denser than purified PMMA. Thus, it is clear that ZrO_2_ can reduce the HVL, making the P-45nZ composite optimal.

The TVL findings are shown as a function of energy in Fig. 10b. The TVL values of P-0Z and P-45nZ samples at the starting energy (i.e., 0.0595 MeV) show a dropping trend from 10.7269 cm to 0.8673 cm because the TVL highly relies on the sample density at all energies. It is evident that decreasing TVL results from the rising density of the composite. The TVL trend depicted in Fig. 10b is consistent with that in Fig. 10a for the HVL. The highest TVL values range from 23.2349 cm for P-45nZ to 33.6143 cm for the P-0Z sample at 1.408 MeV. The high ZrO_2_ content of the P-45nZ sample contributed to its high density and showed the sample’s low TVL. The reverse of the μ values is the MFP values, which are depicted in a manner comparable to that of the HVL and TVL. The smaller the MFP of a composite, the superior the radiation shielding ability. Figure 10c depicts the relationship between the investigated composites’ MFP and the energy. At all energies, the MFP depends on the ZrO_2_ content. Increasing the ZrO_2_ insertion from 0 to 45 wt% in PMMA led to an increase in the density of the samples, from 1.176 for P-0Z to 1.8330 g/cm^3^ for P-45nZ. Consequently, the MFP values drop from 4.6598 for P-0Z sample to 0.3767 cm for P-45nZ at 0.0595 MeV. At higher energy of 1.408 MeV the MFP drops from 14.599 to 9.90 cm. Thus, we can deduce that the P-45nZ sample needs a thinner shielding layer than the other specimens in order to prevent the same radiation, and we can also infer that an increase in energy leads to a rise in the MFP. In conclusion, increasing the content and decreasing the size of ZrO_2_ particles leads to lower values for the HVL, TVL, and MFP parameters, which optimize radiation shielding.

**Figure 11:**
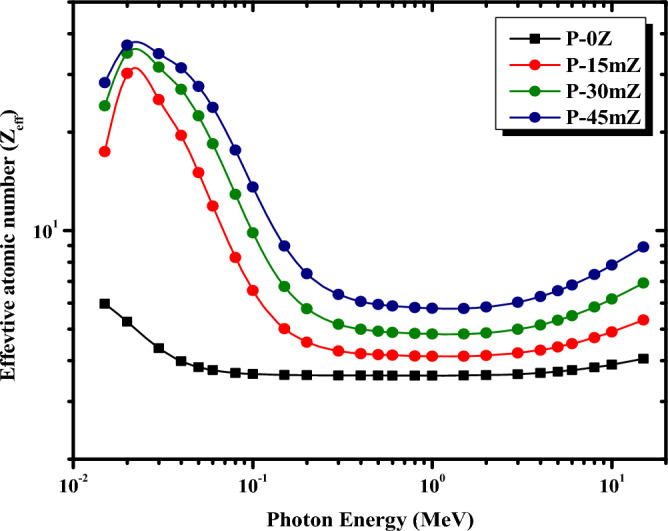
Z_eff_ of pure PMMA, and micro ZrO_2_/PMMA composites at different energies.

**Figure 12:**
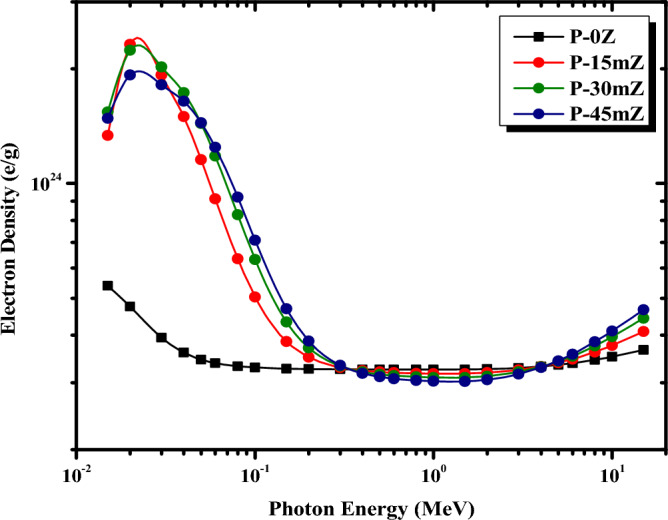
N_eff_ of pure PMMA, and micro ZrO_2_/PMMA composites at different energies.

For the examined pure PMMA and ZrO_2_/PMMA micro composites, the change of Z_eff_ and N_eff_ with photon energy is shown in Figs. 11 and 12, respectively. Evidently, at low energies, the Z_eff_ and N_eff_ reach their maximum values at 0.02 MeV and then decline as the energy increases. This trend can be attributed to the photoelectric process’s cross-section, which is inversely proportional to photon energy as E^3.5^. However, as the photon energy exceeds 0.3 MeV, further increments of photon energy, the value of Z_eff_ becomes virtually independent of photon energy. This behavior might be because the Compton scattering mechanism predominates. At high energies above 1.5 MeV, the value of Z_eff_ slowly rises as the photon energy increases. The supremacy of pair production in this higher energy area can be used to explain this trend. Figure 11 also reveals that, as the concentration of ZrO_2_ filler increases in the PMMA matrix, the values of Z_eff_ increase. This increase is due to the density of ZrO_2_, which increases the overall density of the PMMA-based composites. Therefore, P-45mZ with 45% ZrO_2_ is discovered to have the highest value of Z_eff_ at all γ-ray energies. Eventually, the minimum Zeff corresponds effectively to pure PMMA with 0% of ZrO_2_, which does not contain ZrO_2_ filler. As shown in Fig. 12, N_eff_ exhibits approximately the same behavior as Z_eff_ since the two parameters are strongly linked.

**Figure 13:**
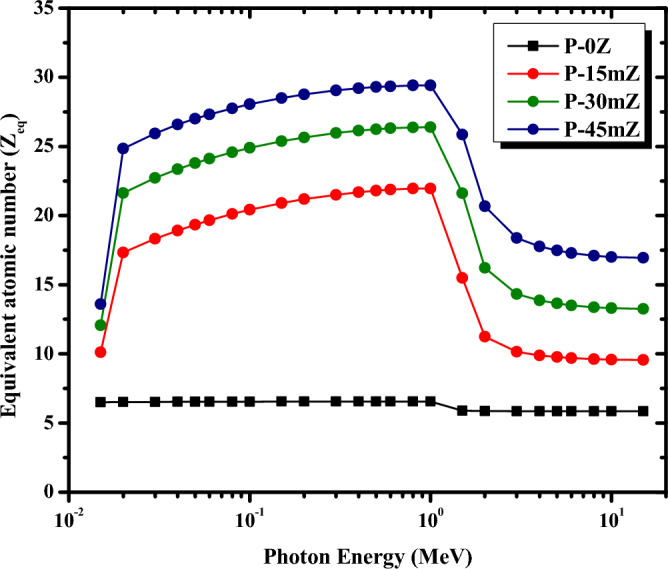
Z_eq_ of pure PMMA, and micro ZrO_2_/PMMA composites at different energies.

The Z_eq_ describes the shielding characteristics of the chosen polymers pertaining to equivalent elements and is also considered when determining the buildup factor. The composites having higher Z_eq_ is the best radiation-protective material. Figure 13 depicts the Z_eq_ values for the micro ZrO_2_/PMMA composites as a function of the photon energy in the range between 0.015 and 15 MeV. From Fig. 13, it is obvious that adding ZrO_2_ in increasing amounts into the PMMA matrix causes the Z_eq_ to increase at the same γ-ray energy. Therefore, the P-0Z sample has the lowest Z_eq_ values, as seen in Fig. 13, whereas the P-45mZ sample has the highest values. Consequently, the P-45mZ composite has better shielding ability than other PMMA composites, which is consistent with the former results of MACs. Furthermore, it is also apparent that the Z_eq_ increases to reach its maximum value for all the ZrO_2_/PMMA composites at 1 MeV due to the Compton scattering (CS) process. The higher observed rise in Z_eq_ values is related to the high rates of CS interaction in the mid-(γ) energy regions, where the Z_eq_ calculation largely depended on the ratio of (MAC_CS_/MAC_total_), implying substantial Compton scattering in the medium energy zone. Then, Z_eq_ drops rapidly as the γ-ray energy exceeds 1.22 MeV due to the pair production process dominating at the higher energy regions.

**Figure 14:**
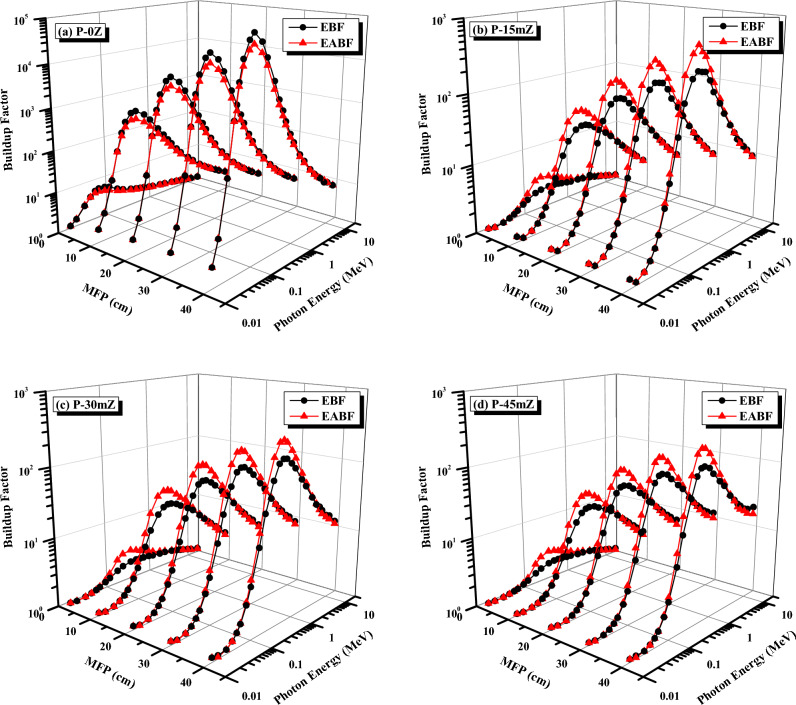
EBF and EABF of P-0Z, P-15mZ, P-30mZ, and P-45mZ as a function of energy at penetration depths of 1, 10, 20, 30, and 40 MFP.

Figure 14 demonstrates the variations of EBF and EABF for P-0Z, P-15mZ, P-30mZ, and P-45mZ samples at various penetration depths as a function of photon energy. It is evident that the EBF and EABF values for the selected composites ascend to a maximal value at middle energies before beginning to fall. The predominant photon interaction mechanism in the low energy region is the photoelectric absorption, whose cross-section changes inversely with energy as E^3.5^. Thus, in this low-energy region, the selected composites can absorb the most photons because of the predominance of this process. Therefore, it causes the EBF and EABF values in the lower energy regions to decrease. On the other hand, pair production, another photon absorption mechanism with a cross-section that is inversely proportional to energy as E^2^, is also predominant in the higher energy area. Compton scattering, a predominant photon interaction process in the intermediate energy region, only reduces photon energy caused by scattering and cannot entirely remove the photon. Because the photon’s lifetime is longer in this energy range, it is more likely to escape from the polymer sample. The values of EBF and EABF are increased as a consequence of this process. Additionally, it is noted that repeated scattering events at large penetration depths cause an increase in the values of EBF and EABF to extremely high levels. It is essential to point out that the variance between EBF and EABF values at the same ZrO_2_ concentration and the same energy is very close. Additionally, a significant decrease in the values of EBF and EABF, accompanied by a shift in their maximum values to higher energies, was observed as the ZrO_2_ content increased.

The variance of EBF and EABF with the radiant energy of all the chosen composites has also been plotted in Fig. 14a–d for certain penetrations depths up to 40 MFP to illustrate the effect of the chemical composition of the selected ZrO_2_/PMMA composites on the EBF and EABF. It is evident that the equivalent atomic number of the chosen polymers has an inverse relationship with the EBF and EABF. Thus, P-0Z, the lowest Z_eq_ polymer, dominates EBF and EABF values at their maximums, while P-45mZ, the greatest Z_eq_ polymer, dominates EBF and EABF values at their minimums. Because P-0Z is a polymer with low-Z components, it could have the highest EBF. Additionally, according to Fig. 14a–d, increasing the thickness of the interacting substance, i.e. increasing the penetration depth of the chosen polymers, causes an increase in the scattering events inside the polymer. Consequently, the EBF and EABF values are incredibly high and display the highest values at the penetration depth of 40 MFP. In light of this, it can be said that P-45mZ has more vital X-ray and γ-ray shielding efficiency than P-0Z.

## Conclusion

In the current study, seven PMMA-based polymer samples were prepared and reinforced with ZrO_2_ MPs and NPs at concentrations of 15, 30, and 45 wt% to examine their radiation shielding capabilities for diverse purposes. The investigated composites are coded as P-0Z, P-15mZ, P-15nZ, P-30mZ, P-30nZ, P-45mZ, and P-45nZ. TEM was used to measure the average size of and ZrO_2_ MPs and NPs. Furthermore, the SEM was used to study the morphology and distribution of ZrO_2_ MPs and NPs within the prepared composites. The analysis revealed that ZrO_2_ NPs had a uniform distribution inside the composites along with a decline in the porosity of the sample in comparison to the ZrO_2_ MPs. The characteristics of ZrO_2_/PMMA molecules were also investigated using FT-IR. The MAC was calculated experimentally using the HPGe detector and five standard radioactive point sources. The experimental results significantly agreed with those obtained theoretically from the XCOM database, indicating the precision of the setup used for computing the MAC for the prepared composites. The experimental findings showed that the prepared samples’ ability to attenuate γ-rays at all the examined energies depends on the size and concentration of ZrO_2_ particles. The findings of this research also demonstrated that PMMA filled with ZrO_2_ NPs has higher μ values than PMMA filled with ZrO_2_ MPs and pure PMMA at all selected energies. P-45nZ sample had the highest μ values, which varied between 2.6546 and 0.0991 cm^−1^ as γ-ray photon energy increased from 0.0595 to 1.408 MeV, respectively. Actually, the MAC for the P-45nZ sample is 1.448 cm^2^/g at 59.5 keV, which is higher than the values reported in the literature and very close to conventional lead at 661.66 keV. Furthermore, the highest relative increase rate in μ values between nano and micro ZrO_2_/PMMA composites was 17.84% reported for the sample P-45nZ at 59.53 keV. The HVL, TVL, and MFP also demonstrated the superiority of ZrO_2_ NPs over ZrO_2_ MPs. Z_eff_ and N_eff_ were increased by increasing the content of ZrO_2_ to the PMMA, which improved the γ-ray shielding efficiency. Due to their easy and quick manufacture, simple processing, non-toxic, lightweight, cost-effective, and environmental friendliness, the proposed composites have advantages over lead materials. Therefore, the developed nano ZrO_2_/PMMA composites are effective shielding materials that can be used to reduce the gamma dose in radiation facilities. Future research could further examine the capability of the proposed composites in shielding neutrons.

## Data Availability

All data generated or analyzed during this study are included in this published article.
